# Decoupling of nutrient element cycles in soil and plants across an altitude gradient

**DOI:** 10.1038/srep34875

**Published:** 2016-10-11

**Authors:** Qiqi Tan, Guoan Wang

**Affiliations:** 1 College of Resources and Environmental Sciences, China Agricultural University, Beijing 100193, China

## Abstract

Previous studies have examined the decoupling of C, N, and P under rapid changes in climate. While this may occur in different environment types, such climactic changes have been reported over short distances in mountainous terrain. We hypothesized that the decoupling of C, N, and P could also occur in response to increases in altitude. We sampled soil and plants from Mount Gongga, Sichuan Province, China. Soil C and N were not related to altitude, whereas soil P increased with altitude. Soil N did not change with mean annual temperature (MAT), mean annual precipitation (MAP), vegetation and soil types, whereas soil P varied with MAT and vegetation type. Plant C remained constant with increasing altitude; plant N exhibited a quadratic change trend along the altitude gradient, with a turning point at 2350 m above average sea level; and plant P decreased with altitude. MAP mostly accounted for the variation in plant P. MAT was responsible for the variation of plant N at elevations below 2350 m, whereas MAT and vegetation type were the dominant influential factors of plants growing above 2350 m. Thus, the decoupling of C, N, and P in both soil and plants was significantly affected by altitude.

Global climate change has severely threatened the existence of various organisms worldwide, and as therefore received much attention in recent years. Because both biological and geochemical processes are affected by climate, climate change also plays an important role in the biogeochemical cycles of elements^1^,^2^,^3^,^4^. Sardans *et al*.^2^ reported, for example, that warming and drought altered C and N concentration, as well as allocation and accumulation in a Mediterranean shrubland. He *et al*.^3^ found that elevated temperature had a negative impact on leaf N and P concentrations of desert plants in China, whereas precipitation had no influence at all. He and Dijkstra^4^ conducted a meta-analysis of plant N and P and concluded that these were negatively affected by drought stress.

The biogeochemical cycles of C, N, and P are coupled in terrestrial ecosystems. This coupling constrains the main processes in ecosystems. It had been suggested, however, that rapid climate change breaks the coupling because of the different controls of climate change on biological and geochemical processes^5^,^6^,^7^,^8^,^9^. Carbon and N cycles are primarily linked to biological processes such as photosynthesis, atmospheric N fixation, and organic matter decomposition. The P cycle, in contrast, is mainly controlled by geochemical processes, such as rock weathering, which is a source of P to terrestrial ecosystems. This has been confirmed by previous research. Ye *et al*.^10^ showed, for example, that a decoupling of above and below ground C and N pools existed in *Stipa grandis* grown along a precipitation gradient in a Chinese steppe zone. Jiao *et al*.^11^ reported that increasing aridity, temperature and soil pH induced soil C-N-P imbalance in grasslands. Elsewhere, Delgado-Baquerizo *et al*.^8^ studied the influence of strengthening aridity on C, N, and P cycles in drylands, and suggested that any predicted increase in aridity would disrupt the coupling of C, N, and P cycles in drylands. This, they claimed, occurred because aridity favoured the dominance of physical processes, such as rock weathering, over biological processes. This was likely to result in a reduction in concentrations of C and N in global drylands, but an increase in P concentration.

In mountainous terrain, changes in climate occur with increasing altitude, even over short distances. We therefore, hypothesized that decoupling of C, N, and P cycles would also occur across an altitude gradient. Although many studies have reported variations in C, N, and P with altitude^12^,^13^,^14^,^15^,^16^,^17^,^18^,^19^,^20^, they did not address changes to the coupling of element cycles associated with altitude. Thus, it remains unclear whether or not this decoupling exists across an altitude gradient. In addition, most previous studies were limited by a narrow altitudinal span, and small species samples.

We selected Mount Gongga in the Sichuan Province of China as our studysite. This site is ideal for examining the influence of climate change on biogeochemical cycles of elements, which is due mostly to its great altitudinal span (from 1100 to 7600 m), abundant species and both intact and varied vertical vegetation belts.

Element cycles in soil and plants are the main components of biogeochemical cycles in terrestrial ecosystems. Delgado-Baquerizo *et al*.^8^ addressed the influence of climate change on the decoupling of C, N, and P in soil; whereas Yuan and Chen^9^ focused on this decoupling in plants. To our knowledge, no studies have described the decoupled relationship of C, N, and P in both soil and plants at the same time. In order to gain a better understanding of the influence of climate change on C, N, and P cycles in terrestrial ecosystems, element cycles in both soil and plants should be studied.

The study we conducted on Mount Gongga addressed the C, N, and P cycles in not only soil, but plants as well. Our objective was to examine whether or not the biogeochemical cycles of C, N, and P were uncoupled across an altitude gradient. In order to test our hypothesis, we investigated nutrient variations in both soil and plants across an altitude gradient that spanned 3300 m, and explored the influence of climate change on soil and plant stoichiometry associated with altitude. We now report the results of our study.

## Results

### Changes of C, N, and P in both soil and plants across an altitudinal gradient

**Figure 1:**
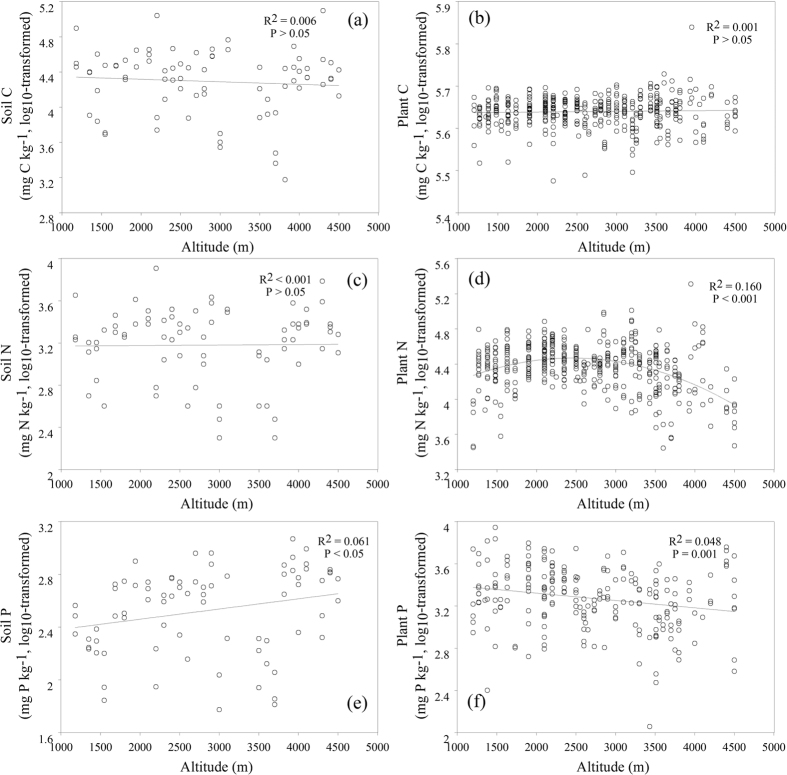
Changes to C, N, and P in soil and plants associated with altitude. (**a**) soil C, (**b**) plant C, (**c**) soil N, (**d**) plant N, (**e**) soil P, (**f**) plant P.

Soil C, N, and P on Mount Gongga ranged from 1.5 to 125.4 mg · g^−1^, 0.2 to 8.1 mg · g^−1^ and 0.03 to 1.2 mg · g^−1^, with an average of 26.4 mg · g^−1^, 2.0 mg · g^−1^ and 0.4 mg · g^−1^, respectively. The variability in soil C and N did not exhibit a clear trend with altitude (p > 0.05 for both) (Fig. 1a,c), whereas soil P increased significantly across the altitudinal gradient (p < 0.05) (Fig. 1e).

Plant C, N, and P on Mount Gongga ranged from 298.9 to 690.1 mg · g^−1^, 2.8 to 59.1 mg · g^−1^ and 0.1 to 8.8 mg · g^−1^, with an average of 437.4 mg · g^−1^, 22.2 mg · g^−1^ and 2.3 mg · g^−1^, respectively. Plant C remained constant with altitude (p > 0.05) (Fig. 1b). Plant N first increased with increasing altitude and then decreased (p < 0.001), with the inflection point of the curve occurring at 2350 m. This was based on segmented regression (Fig. 1d). The plant P decreased with increasing altitude (p = 0.001) (Fig. 1f).

### Effects of soil type and vegetation type on soil and plant elements vs. altitude

The C concentrations in soil and plants remained constant with increasing altitude. Previous results had also reported the same pattern seen in this study, and suggested that plant C was not related to climate and environmental conditions^21^,^22^,^23^. In natural ecosystems, soil C is mainly derived from plant C. Soil C concentration on Mt. Gongga also remained constant across the altitude gradient and was independent of climate and environment conditions. We did not, therefore, perform any statistical analyses to determine the relationships between C in soil and plants and climate.

Partial correlation analyses between soil N and altitude, after controlling for vegetation type, soil type or the combined effects of vegetation and soil types, revealed no clear altitudinal trends when it came to soil N. Partial correlation analyses yielded almost the same results as a bivariate correlation analysis between soil N and altitude (Table 1). This suggested that the pattern of soil N versus altitude was independent of vegetation type and soil type. The relationship between soil P and altitude shown in Fig. 1 did not, however, remain as vegetation type, soil type or the combined effects of vegetation and soil types, for which they were controlled for (Table 1). This indicated that vegetation and soil type affected the relationship between soil P and altitude.

Although the vegetation type remained constant from 2200–2800 m, at an altitude of 2350 m there was an inflection point of the altitudinal variation in plant N (Fig. 1d), as identified by segmented regression. Since the altitudinal trend in plant N changed at <2350 m and >2350 m, the main influential factors for the plants growing below 2350 m might differ from those for plants occurring above 2350 m. We therefore performed the statistical analyses for plant N both below and above 2350 m.

Since there was no change to soil type below 2350 m, only vegetation type was controlled for in the partial correlation analysis of plant N (<2350 m) vs. altitude. It yielded almost the same results as the bivariate correlation analysis of plant N (<2350 m) vs. altitude (Table 2). This suggested that vegetation type exerted no influence on the pattern of plant N vs. altitude since the altitude was below 2350 m. The relationship between plant N (>2350 m) and altitude remained constant when vegetation type, soil type or the combined effects of vegetation and soil types were taken into consideration (Table 2). This suggested that neither vegetation nor soil type exerted any influence on the pattern of plant N vs. altitude (>2350 m).

Since plant P was linearly related to altitude without an inflection point, we did not deem it necessary to conduct a statistical analysis of plant P below and above 2350 m. Plant P was no longer related to altitude since vegetation type, or the combined effects of vegetation and soil types, were controlled for, the relationship between plant P and altitude was influenced by soil type to some extent, even though this relationship remained constant (Table 2). This suggested that the pattern of plant P vs. altitude depended on vegetation type, and soil type also had an influence on it.

### Influences of MAT, MAP, vegetation type and soil type on soil nutrients

Multiple regression using MAT and MAP as independent variables, and soil N as the dependent variable, revealed that only 3.6% of the variability in soil N could be explained as a linear combination of both environmental factors (R^2^ = 0.036, p = 0.490) (Table 3). In addition to the effects of quantifiable environmental factors, qualitative factors, such as soil type and vegetation type, might also influence on soil N. In order to further constrain the effects of soil and vegetation types on soil N, multiple regressions with these as dummy variables were conducted. Given the strong relationship between soil and vegetation types, the soil and the vegetation variables were separately introduced into the statistical analyses. Compared to the multiple regressions in which only quantitative environmental variables were introduced, multiple regressions with vegetation, exhibited greater variance (R^2^ = 0.086, p = 0.562) (Table 3). Multiple regressions, in which soil type was also introduced, did not reveal any variability in soil N (R^2^ = 0.039, p = 0.621) (Table 3). This suggested that vegetation type exerted a slight effect on soil N. The MAT and MAP in total contributed little to the variance in soil N, whereas soil type made no contribution.

The combined contribution of MAT and MAP to variability in soil P was 11.5% (R^2^ = 0.115, p = 0.043) (Table 3). Considering there was the possibility of correlations among the two explanatory variables, stepwise regression was used to eliminate the potential influence of collinearity between them. Variables were incorporated into the model with *P*-value < 0.05. These were excluded if *P*-value > 0.1. Therefore, the stepwise regression of soil P consisted only of MAT (R^2^ = 0.071, p = 0.025). When vegetation type was taken into consideration, the degree of explanation of the variability in soil P increased to 20.9% (R^2^ = 0.209, p = 0.035) (Table 3). When soil type was incorporated into the model, however, the R^2^-value increased, but was not significant (R^2^ = 0.129, p = 0.058) (Table 3). It suggested that both MAT and vegetation type played a significant role in soil P, whereas soil type had no influence.

### Influences of MAT, MAP, soil nutrients, vegetation type and soil type on plant nutrients

Considering that the nutrient source of plants was mainly derived from soil, soil nutrients were factored into the explanation of the variability in plant nutrients. Multiple regression analyses were performed with MAT, MAP, and soil nutrients (soil C, soil N, and soil P) used as independent variables, whereas plant N (<2350 m) used as the dependent variable. The results showed that 13.2% of the variability in plant N (<2350 m) could be explained by the linear combination of all five factors (R^2^ = 0.132, p = 0.002) (Table 4). Stepwise regression, in which the five variables were introduced, showed that the major influential factors of plant N (<2350 m) was MAT (R^2^ = 0.123, p < 0.001). When vegetation type was also taken into account, the degree of explanation of plant N variability (<2350 m) did not change (R^2^ = 0.132, p = 0.002). It suggested that vegetation type had no impact on plant N (<2350 m) (Table 4).

The combined contribution of MAT, MAP and soil nutrients to variability in plant N (>2350 m) was 37.2% (R^2^ = 0.372, p < 0.001) (Table 4). The most influential factor was MAT (R^2^ = 0.371, p < 0.001). This was based on stepwise regression in which MAT, MAP, and soil nutrients were introduced. When vegetation type was incorporated into the model, it revealed more variance in plant N (>2350 m) (R^2^ = 0.481, p < 0.001) (Table 4), even though the R^2^-value did not alter when soil type was incorporated into the model (R^2^ = 0.372, p < 0.001) (Table 4). It indicated that vegetation type also exerted a significant impact on plant N (>2350 m), whereas soil type had no influence.

The MAT, MAP and soil nutrients explained 27% of the variability in plant P (R^2^ = 0.270, p < 0.001) (Table 4). Stepwise regression showed that precipitation mainly influenced plant P (R^2^ = 0.242, p < 0.001). When vegetation type was introduced into the model, the degree of explanation of the variability in plant P increased (R^2^ = 0.281, p < 0.001) (Table 4), but rarely changed when soil type was taken into account (R^2^ = 0.274, p < 0.001) (Table 4). It suggested that both vegetation type and soil type contributed little to plant P.

## Discussion

This study showed that soil N did not vary with altitude (Fig. 1c), and that the pattern of soil N vs. altitude was independent of vegetation type and soil type. This was based on partial correlation analyses in which vegetation and soil type were controlled for (Table 1). This suggested that no relationship existed between soil N and altitude. This may be a pattern not only for soils on Mount Gongga, but also for those of other mountainous terrains. The lack of variance in soil N with increasing altitude (1400–3100 m) was also reported for Mt. Teide (Canary Islands)^15^. In contrast, Zhang *et al*.^24^ and Shedayi *et al*.^23^ reported a positive correlation between soil N and altitude (3000–4000 m and 2860–3500 m, respectively); Yang *et al*.^25^ showed that soil N was lower at higher elevations than lower elevations (2000–2600 m) in the Changbai Mountain Range between China and North Korea. Limited altitude ranges, as well as gaps in the data of the studies by Zhang *et al*.^24^, Shedayi *et al*.^23^, and Yang *et al*.^25^ may have prevented a real pattern from emerging. Köhler *et al*.^15^ suggested that lack of altitudinal change in soil N at Mt. Teide was because the local soil is poorly developed volcanic regosols with low cation exchange capacity and comparably low soil N. Obviously, this reason could not be applied to Mount Gongga, because the soils on Mount Gongga are not of a volcanic nature. Furthermore, the soil N (0.02 to 0.81%) on Mount Gongga was greater than that reported in Köhler *et al*.^15^ (0.06 to 0.26%). We believed that no altitudinal variation in soil N was the result of competition among the two mechanisms, i.e., microbe N-fixation and plant N-uptake.

At low elevations, high temperatures increase high activity in soil enzymes, thus resulting in high microbe N-fixation. Higher temperatures also result in greater plant N-uptake. The opposite occurs at high altitudes. Thus, no altitudinal variation in soil N suggested there was biological resistance to environmental change, and that climate change associated with altitude had no influence on the variance in soil N. This was further confirmed by multiple regression analysis, which showed that MAT and MAP did not significantly contribute to the variability in soil N (Table 3).

Our study also showed there was a positive relationship between soil P and altitude (Fig. 1e), and that this relationship was dependent on vegetation and soil types (Table 1). It suggested that the positive correlation between soil P and altitude might be a local phenomenon. Zhou *et al*.^19^ also reported a positive altitudinal variance in soil P on Mount Gongga. In contrast, Yang *et al*.^25^ showed that soil P was greater at lower elevations than higher ones in Changbai Mountain. Since the study by Yang *et al*.^25^ was conducted in an alpine tundra ecosystem, unlike the diverse ecological systems of our study, it could explain the discrepancy between our studies.

Multiple regression analysis showed that the main influential factors of soil P were MAT and vegetation type (Table 3). Bing *et al*.^20^ also found that soil P depended on vegetation type on Mount Gongga. Although MAP was not used in the model of stepwise regression, it played a significant role in soil P. Since precipitation is associated with temperature, there was a strong collinearity between them. On Mount Gongga, as altitude increased, temperature significantly decreased, whereas precipitation increased significantly. (The annual precipitation on Mount Gongga exceeds 2000 mm above 3000 m) (Table S1). This combination of lower temperature and higher precipitation accelerates rock weathering through a strong freeze-thaw cycling. More soil P therefore accumulates at high elevations because of the weathering.

Previous studies have suggested that decoupled C, N, and P cycles occur in soil during rapid environmental change^5^,^8^. We found that soil C and N remained constant, whereas soil P increased with altitude. This suggests a decoupling of C, N, and P associated with altitude, and the decoupling was due to different effects of altitudinal climate change on biological and geochemical processes.

Previously, we reported that plant N was non-linearly correlated with altitudinal gradients on Mount Gongga^26^. In this present study, we were able to determine the inflection point at which altitudinal variation in plant N occurred. This was established by the segmented regression analysis. The partial correlation analyses revealed that the pattern of altitudinal variation in plant N was independent of vegetation or soil types (Table 2). This indicated that the non-linear variation in plant N due to altitude might be a common phenomenon that not only exists on Mount Gongga, but elsewhere, too.

This study also revealed influential factors pertaining to plant N. The major influential factor was MAT for those plants growing at altitudes both below and above 2350 m. This was based on stepwise regression. Shi *et al*.^26^ suggested that the quadratic variation of plant N across the altitude gradient might be the result of the competition of two mechanisms, the temperature-plant physiological hypothesis (TPPH) and the biogeochemical hypothesis (BH). The TPPH suggests that plants increase their nutrient concentration to offset the suppression of their physiological rates caused by low temperature^27^. As a consequence, plant N increases with altitude. The BH suggests that soil nutrient mineralization decreases under low temperature conditions, thus decreasing soil nutrient availability. As a result, the amount of nutrient by plant uptake also decreases^27^; this leads to a decrease in plant N with altitude. In the present study, the influence of TPPH exceeded that of BH at an elevation below 2350 m. For the plants growing at altitudes above 2350 m, BH was more influential than TPPH. A MAT of 8.8 °C was the temperature at which leaf N vs. altitude changed. Reich and Oleksyn^27^ showed that plant N decreased with temperature when MAT was greater than 5–10 °C, however, it increased with temperature when MAT was lower than 5–10 °C. Kang *et al*.^28^ also reported a similar relationship between leaf N and MAT for *Picea abies*; their study showed that the peak of N concentration occurred at 6–8 °C, and this trend was observed across Europe. Thus, we suggest that the nonlinear relationship of leaf N-MAT might be a general pattern.

In addition to MAT, multiple regression analysis showed that vegetation type also influenced N variation in plants growing above 2350 m (Table 4). Communities with similar life-forms of dominant plants were generally considered as the same vegetation type. Thus, the dominant plant species within the same vegetation type generally belonged to the same functional group. It has been suggested that plant N varies significantly across functional groups^21^,^29^,^30^,^31^,^32^,^33^,^34^. This results in vegetation type control of plant N. For those plants occurring below 2350 m, multiple regression analysis did not reveal any influence of vegetation type on plants N (Table 4). Only one vegetation type occurred at elevations below 2200 m, possibly accounting for this result.

The results of this study also show that plant P was negatively correlated to altitude (Fig. 1). This negative relationship was reported in most previous studies^14^,^15^,^16^,^17^,^35^,^36^,^37^. However, an increase in plant P due to increased altitude was also observed^12^,^21^,^38^,^39^. Multiple regression analysis suggested that neither vegetation nor soil type had any influence on leaf P (Table 4). Stepwise regression revealed that precipitation controlled leaf P. Given that accelerated rock weathering at high elevations results in increased P levels in soils, precipitation increases soil P leaching. The diffusion of soil P into the soil solution was, however, decreased^40^. Available phosphorus (e.g. phosphate) might therefore decrease with increasing precipitation and altitude. Furthermore, low temperature limits root uptake. The combined influence of these processes may account for the decrease in plant P due to altitude.

In addition to the afore-mentioned mechanisms accounting for altitudinal variances in leaf N and P, leaf morphology may explain some of the variability observed. Several studies have reported that leaf traits, such as leaf area, thickness, density, leaf mass per area (LMA) and specific leaf area (SLA), varied with altitude^12^,^41^,^42^,^43^,^44^,^45^. For example, Cordell *et al*.^44^ showed that *Metrosideros polymorpha*, the dominant tree species in Hawaiian forest ecosystems, experienced increased leaf LMA with higher elevations. Körner^12^ and Körner *et al*.^41^ reported greater leaf thickness with increased altitude. Körner *et al*.^42^ suggested that climate variables were responsible for the altitudinal trends in leaf morphology.

Leaf morphology has, however, been correlated with nutrient concentration. Cordell *et al*.^44^ revealed there was a positive relationship between LMA and area-based leaf N. Kloeppel *et al*.^43^ found that mass-based leaf N concentration was positively correlated to SLA for both larch and evergreen conifers. Thus, altitudinal trends in leaf nutrients may be linked to variations in leaf morphology. Since leaf traits were not measured in this study; altitudinal variations in leaf nutrients on Mount Gongga could not be explained in terms of leaf morphology. Leaf morphology should be considered in future studies.

Yuan and Chen^9^ concluded that the decoupling of N and P occurred in terrestrial plants undergoing climate change. This study showed that increasing altitude had no effect on plant C; N first increased and then decreased, and P decreased. Our study suggested decoupled C, N, and P in plants occurred with increasing altitude. The altitudinal variations in plant C and N indicated that there was biological resistance of plants to environment change. The variation in plant P may be controlled by freeze-thaw cycling, i.e. a geological process. In short, different controls of biological and geochemical processes on plant C, N, and P have resulted in the decoupling of C, N, and P in plants in a mountainous terrain.

## Conclusion

To our knowledge, this study was the first to discuss the decoupled relationship of C, N, and P in both soil and plants across an altitude gradient. Our results suggest a decoupling of C, N, and P in both soil and plants associated with altitude. Although this study was conducted in mountain terrain, mountainous climate can significantly change over a short distance, thus, the findings of this study could help understand the soil and plant stoichiometric traits in response to global climate change. It is expected based on our findings that soil C and N were independent of any predicted warming, whereas soil P decreases with increasing temperature. Plant C is likely to remain stable under warming conditions. The influence of global warming on plant N would depend on local temperature. Areas where MAT was approximately >8.8 °C are likely to experience a decrease in plant N with increasing temperature, whereas the opposite is likely to occur in areas where MAT was approximately <8.8 °C. The accumulation of P in plants would be limited by increases in precipitation.

## Methods

### Study site

Mount Gongga is located on the southeastern side of the Qinghai-Tibet Plateau, China (101°30′~102°10′E, 29°20′~30°00′N). It was selected as the study site for this research because its eastern slope consists of different climate types, diverse ecological systems, stable vegetation types ranging from tropical and subtropical to cold zone, and relatively little human disturbance. The altitude along the eastern slope varies from 1100 m above average sea level (a.s.l.) to 7600 m a.s.l., also making it desirable. There are three meteorological observatories on the eastern slope. Meteorological data from the three observatories shows that the climate is warm and dry at low elevations and cold and moist at high elevations. Temperature decreases with increasing altitude, whereas precipitation increases (Table S1)^46^.

There is an intact and continuous vertical vegetation spectrum along the eastern slope of Mount Gongga. It consists of the following vegetation types: subtropical evergreen broad-leaved vegetation (1100–2200 m, including a semi-arid valley with shrubs and grasses (<1500 m), evergreen broad-leaved forests, and deciduous broad-leaved forests); temperate coniferous and broad-leaved mixed forests (2200–2800 m); frigid dark coniferous forests (2800–3600 m); alpine subfrigid shrub and meadow vegetation (3600–4200 m); alpine frigid meadow vegetation (4200–4600 m); alpine frigid sparse grasses and a desert zone (4600–4800 m), and a high-altitude alpine ice-and-snow zone (>4900 m). The vertical distribution of soil on the eastern slope of Mount Gongga is significant. A continuous soil sequence occurs from 1100 m to 4900 m. It consists of luvisols (1100–3600 m), cambisols (3600–4600 m) and cryosols (>4600 m).

### Field sampling

**Figure 2:**
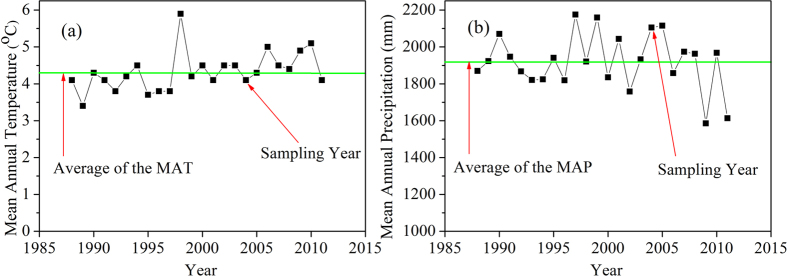
Mean annual temperature (MAT) and mean annual precipitation (MAP) ranging 1988−2011. (**a**) MAT and (**b**) MAP. Note that all data were derived from the Hailuogou ecological observatory at 3000 m.

A vertical transect was laid out on the eastern slope of Mount Gongga (from 1200 m a.s.l. to 4500 m a.s.l.). Soil and plant samples were collected along that transect at intervals of approximately 100 m in August 2004. Figure 2 shows the MAT and MAP data collected at the Hailuogou meteorological observatory (3000 m a.s.l.), spanning from 1985 to 2011. The climactic conditions of this sampling year could be regarded as a typical year, despite the MAP being somewhat higher relative to the mean MAP from 1985 to 2011. Sampling was restricted to unshaded sites and as far from human habitats as possible. Almost all species which could be found at each sampling altitude were collected. At each site, 5–7 plants of each species of interest were identified, and the same number of leaves was sampled from each individual. The uppermost leaves of each individual were collected. For tree species, two leaves at each of the four cardinal directions (eight leaves in total) were collected from each individual, which were from positions of full-irradiance approximately 8–10 m above the ground. Leaves from each species at each elevation were mixed into one sample for further measurement. There were a total of 444 plant samples, including 12 fern samples and 432 seed plant samples, collected on Mount Gongga. A total of 9 fern species and 269 seed plant species were included in these samples. Among the 269 seed plant species, only 7 samples (5 species) were N-fixing plants. All of them occurred below 1900 m a.s.l.

At most sampling sites, we set three plots (0.5 m × 0.5 m) within a 200 m^2^ area. All aboveground litters within the plots were removed, after which a soil profile was dug into the weathered rock. In total, 75 soil profiles were sampled along the transect. Organic layers above mineral soil were defined as “litter.” Depth zero refers to the top of the mineral horizon. Mineral soil was collected at 5 cm intervals down to a 10 cm depth, after which it was sampled at 10 cm intervals down to the bottom of the soil profile.

### Measurements of C, N, and P in soil and plants

Soil samples (0–5 cm depth) were air-dried in the field, followed by oven drying at 70 °C until a constant weight was achieved. These were then ground into fine powder after picking out stones and plant residue. Plant samples were also air-dried in the field and oven-dried at 70 °C, and also ground into fine powder. The C and N concentrations of soil and plants were measured using an elemental analyzer (Flash EA1112, CE Instruments, Wigan, UK) in the Stable Isotope Laboratory of the College of Resources and Environmental Sciences, China Agricultural University. Standard deviations for the measurements of C and N concentrations were 0.1%. The methods of total P measurements for plants and soil used in the present study were based on Lu (2000)^47^. P of plants was analysed by the molybdate/ascorbic acid method after H_2_SO_4_-H_2_O_2_ digestion. The absorbance of each sample was measured at 700 nm after adding molybdenum-stibium-ascorbic acid reagent. The colorimetric time was 30 min, whereas the colorimetric temperature was greater than 15 °C. Soil total P was measured by the same method as plant total P after H_2_SO_4_-HCLO_4_ digestion. The C, N, and P concentrations of soil and plant samples were expressed as mg/kg on a dry mass basis. Because total P in plants was measured after the C and N measurements, the size of many plants were not enough for the P analysis. Data of plant P (222 samples in total) was less than that of plant C and N (444 samples in total).

### Data analysis

Since there were only three meteorological observatories on the eastern slope of Mount Gongga, meteorological data (MAT and MAP) for the other sampling altitudes were calculated using a linear interpolation method, based on data derived from the three observatories^46^. The variation gradient of MAP with altitude from 1000 m to 2000 m, 2000 m to 3600 m and 3600 m to 5000 m are 120 mm/100 m, 74 mm/100 m and 66 mm/100 m, respectively. The gradient of MAT from 1000 m to 5000 m was approximately 0.6 °C/100 m. The C, N, and P of soil and plant samples were log_10_-transformed before regression analyses to improve data normality. Regression analyses of C, N, and P in soil and plants against altitude were performed to address the altitudinal pattern of nutrient elements. Segmented regression was used to determine the inflection point of the altitudinal variation in plant N. Partial correlation analyses were used to detect the effects of soil type and vegetation type on soil and plant traits vs. altitude. Multiple regressions of soil nutrients against MAT, MAP, vegetation type, and soil type were used to explore the influences of climate, vegetation type and soil type on soil nutrients. Multiple regressions of leaf nutrients against MAT, MAP, soil C, soil N, soil P, vegetation type, and soil type were conducted to reveal the influences of climate, soil nutrients, vegetation type and soil type on plant nutrients. Stepwise regression was performed to investigate the major influential factors of soil and plant nutrients when the multiple regression model was significant. All statistical analyses were performed using SPSS20.0 (IBM Corporation, USA), with significance being determined at the 0.05 level.

## Additional Information

**How to cite this article**: Tan, Q. and Wang, G. Decoupling of nutrient element cycles in soil and plants across an altitude gradient. *Sci. Rep.* **6**, 34875; doi: 10.1038/srep34875 (2016).

## Supplementary Material

Supplementary Information

## Figures and Tables

**Table 1:** Bivariate correlation between soil nutrients and altitude, and the partial correlations of soil nutrients and altitude as vegetation type, soil type and the combined influence of vegetation and soil types were controlled for, respectively.

Soil nutrients	Variable controlled for	*r*	*p*
N	None^†^	0.013	ns
	Vegetation type^#^	0.003	ns
	Soil type^$^	0.056	ns
	Combined influence^&^	0.014	ns
P	None^†^	0.225	*
	Vegetation type^#^	0.147	ns
	Soil type^$^	0.159	ns
	Combined influence^&^	0.138	ns

Note: r is correlation coefficient; p is significance of correlation analysis; * and ns means p < 0.05 and p > 0.05, respectively; ^†^is the bivariate correlation between soil nutrients and altitude; ^#, $^ and ^&^ represent the partial correlations of soil nutrients and altitude as vegetation type, soil type and the combined influence of vegetation and soil types were controlled for, respectively.

**Table 2:** Bivariate correlation between plant nutrients and altitude, and the partial correlations of plant nutrients and altitude as vegetation type, soil type and the combined influence of vegetation and soil types were controlled for, respectively.

Plant nutrients	Variable controlled for	*r*	*p*
N (<2350 m)	None^†^	0.373	***
	Vegetation type^#^	0.414	***
N (>2350 m)	None^†^	−0.347	***
	Vegetation type^#^	−0.182	**
	Soil type^$^	−0.328	***
	Combined influence^&^	−0.182	**
P	None^†^	−0.221	**
	Vegetation type^#^	−0.111	ns
	Soil type^$^	−0.153	*
	Combined influence^&^	−0.120	ns

Note: r is correlation coefficient; p is significance of correlation analysis; *^,^ **^,^ *** and ns means p < 0.05, p < 0.01, p < 0.001 and p > 0.05, respectively; ^†^is the bivariate correlation between soil nutrients and altitude; ^#, $^ and ^&^ represent the partial correlations of soil nutrients and altitude as vegetation type, soil type and the combined influence of vegetation and soil types were controlled for, respectively. Soil type did not change below 2350 m, thus, only vegetation type was controlled for in partial correlation analysis of plant N (<2350 m) vs. altitude.

**Table 3:** Results from multiple regressions of soil nutrients against MAT, MAP, vegetation type and soil type.

Soil	Model-1			Model-2			Model-3		
	R^2^	Adjusted R^2^	p	R^2^	Adjusted R^2^	p	R^2^	Adjusted R^2^	p
N	0.036	−0.008	0.490	0.086	−0.017	0.562	0.039	−0.020	0.621
P	0.115	0.075	0.043	0.209	0.119	0.035	0.129	0.075	0.058

Note: Model-1, Model-2 and Model-3 are the multiple regressions of soil nutrients against MAT and MAP, against MAT, MAP and vegetation type, and against MAT, MAP and soil type, respectively.

**Table 4:** Results from multiple regressions of plant nutrients against MAT, MAP, soil nutrients (soil C, N, and P), vegetation type and soil type.

Plant	Model-4			Model-5			Model-6		
	R^2^	Adjusted R^2^	p	R^2^	Adjusted R^2^	p	R^2^	Adjusted R^2^	p
N (<2350 m)	0.132	0.099	0.002	0.132	0.099	0.002			
N (>2350 m)	0.372	0.340	0.000	0.481	0.444	0.000	0.372	0.336	0.000
P	0.270	0.224	0.000	0.281	0.214	0.000	0.274	0.223	0.000

Note: Model-4 is the multiple regressions of plant nutrients against MAT, MAP, soil C, soil N, and soil P. Model-5 is the multiple regressions of plant nutrients against MAT, MAP, soil C, soil N, soil P, and vegetation type. Model-6 is the multiple regressions of plant nutrients against MAT, MAP, soil C, soil N, soil P, and soil type. Model-6 was not performed for the plant N below 2350 m due to the soil type was same below the altitude.
