# Supplementary material for: Decoupling of nutrient element cycles in soil and plants across an altitude gradient
Source: Sci Rep. 2016 Oct 11;6:34875. doi: 10.1038/srep34875 (PMC5057141; doi:10.1038/srep34875)
Supplement: Supplementary Information [file srep34875-s1.pdf]

Title

Decoupling of nutrient element cycles in soil and plants across an altitude gradient

Qiqi Tan & Guoan Wang*

College of Resources and Environmental Sciences, China Agricultural University, Beijing 100193, China

Author for correspondence:

Guoan Wang

Tel: +086-10-62733942

Email:gawang@cau.edu.cn

Table S1 The climate information (1960–1990) derived from three meteorological observatories on the east slope of Mount Gongga.

Meteorological observatories	Detuo	Moxi	Hailuogou ecological observatory
Altitude (m)	900	1640	3000
MAP (mm)	700	1050	1938
MAT (℃)	16.6	12.2	4.0
